# Shifting Acetylene Hydrochlorination From the Gas to the Liquid Phase: Vinyl Chloride Production in Bichloride‐Based Ionic Liquids

**DOI:** 10.1002/advs.202503992

**Published:** 2025-06-10

**Authors:** Gesa H. Dreyhsig, Vera Giulimondi, Merlin Kleoff, Letizia Lanfredi, Maxim Fuchs, Niklas Limberg, Marc Reimann, Martin Kaupp, Javier Pérez‐Ramírez, Sebastian Riedel

**Affiliations:** ^1^ Institute of Chemistry and Biochemistry Inorganic Chemistry Freie Universität Berlin Fabeckstr. 34/36 14195 Berlin Germany; ^2^ ETH Zürich Department of Chemistry and Applied Biosciences Institute of Chemical and Bioengineering Vladimir‐Prelog‐Weg 1 Zürich 8093 Switzerland; ^3^ Institue for Chemistry Theoretical Chemistry Technische Universität Berlin Straße des 17. Juni 135 10623 Berlin Germany

**Keywords:** catalysis, hydrogen bonds, Ionic liquids, palladium, vinyl chloride synthesis

## Abstract

Herein, [NEt_3_Me]_2_[PdCl_4_] is reported as a highly active catalyst for the mercury‐free hydrochlorination of acetylene to vinyl chloride, resulting from the combination of the bichloride‐based ionic liquid [NEt_3_Me][Cl(HCl)*_n_*] with PdCl_2_. Replacing gaseous HCl with the bichloride shifts the reaction in the liquid phase increasing the process safety by pressure reduction and achieves a turnover frequency of TOF = 110 mol_VCM_ h^−1^ mol_PdCl2_^−1^ matching the productivity of state‐of‐the‐art heterogeneous systems. Additionally, [NEt_3_Me]_2_[PdCl_4_] shows remarkable long‐term stability and can be re‐used over ten reaction cycles (200 h in total) without any problems due to its resistance to reduction by acetylene and coking as revealed by kinetic, theoretical, and spectroscopic investigations. Finally, initial catalytic studies demonstrate promising outcomes for applications of the bichloride‐based ionic liquids with other noble metals, like platinum, if disproportionation phenomena, as observed for gold, do not occur.

## Graphical abstract

Combining the bichloride‐based ionic liquid [NEt_3_Me][Cl(HCl)*_n_*] with PdCl_2_ results in the formation of the highly active, mercury‐free catalyst [NEt_3_Me]_2_[PdCl_4_] for acetylene hydrochlorination to vinyl chloride. Replacing HCl with the bichloride improves the process safety and achieves a competitive turnover frequency of 110 mol_VCM_ h^−1^ mol_PdCl2_^−1^. The system is stable and re‐usable, with a potential for other applications.

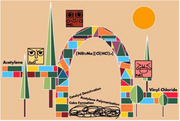

## Introduction

Due to the local abundance of coal and therefore acetylene in China, one third of the world's vinyl chloride monomer (VCM) demand is produced by the hydrochlorination of acetylene with hydrogen chloride (HCl).^[^ ^1^, ^2^, ^3^, ^4^ ^]^ This reaction typically runs on carbon‐supported HgCl_2_ catalysts and needs special safety equipment and devices to handle gaseous HCl.^[^ ^2^, ^4^, ^5^ ^]^

Since the employed carbon‐supported mercury‐based catalysts suffer from high volatility leading to deactivation and posing environmental and health hazards, their use is demanded to be phased out in industrial processes by the Minamata Convention and more stable and sustainable alternatives are urgently required.^[^ ^2^, ^4^, ^6^, ^7^, ^8^ ^]^ The work of Hutchings, Pérez–Ramírez, and coworkers on heterogeneous gold and platinum catalysts, respectively, have shifted the focus to noble metals, achieving high performance upon nanostructuring Au(i)Cl and Pt(ii)Cl_x_ (x = 2‐3) as isolated cationic single atoms on activated carbon.^[^ ^1^, ^4^, ^6^, ^9^, ^10^, ^11^, ^12^, ^13^, ^14^ ^]^ Besides gold and platinum, also carbon‐supported palladium – as cationic Pd(ii) species – shows a high initial catalytic activity for the hydrochlorination of acetylene but suffers from a fast deactivation due to coke formation.^[^ ^15^, ^16^, ^17^, ^18^, ^19^, ^20^ ^]^ Accordingly, several other efforts have been undertaken to stabilize Pd catalysts using, e.g., functionalized carbon materials or HY zeolites.^[^ ^17^, ^18^, ^19^, ^20^, ^21^, ^22^ ^]^

Especially ionic liquids (ILs) have been found to effectively stabilize Pd‐based catalysts as so‐called Supported‐Ionic‐Liquid‐Phases (SILPs) and to prevent the reduction of catalytically active Pd(ii) to Pd(0).^[^ ^18^ ^]^ Additionally, their use allows performing reactions in a liquid environment that offers not only a low vapor pressure and good thermal stability but also an efficient heat dissipation. This avoids local heat accumulations associated with detrimental coke formation by acetylene polymerization and catalyst deactivation.^[^ ^2^, ^17^, ^23^ ^]^ Furthermore, ILs could also influence the structure and thus the reactivity of the catalytically active species.^[^ ^19^, ^20^, ^21^, ^22^, ^23^, ^24^, ^25^ ^]^

Although these works on new, stable, and efficient catalytic systems enable the mercury‐free hydrochlorination of acetylene, they still require the use of pure HCl. Hydrogen chloride is a highly toxic and corrosive gas building up a vapor pressure of 42.6 bar at 20 °C when pressure liquefied.^[^ ^26^ ^]^ This makes its handling and storing rather difficult and requires materials with resilience and specially trained staff.^[^ ^5^ ^]^ However, failure of the facility and unintentional release of HCl can never be fully excluded and incidents like at Wacker Polysilicon North America in Charleston, Tennessee, are often fatal.^[^ ^27^ ^]^ To address this, Riedel and coworkers recently introduced easy‐to‐handle poly‐ and bichloride‐based ILs to safely store chlorine and HCl, respectively. Even at higher temperatures, both systems show almost no vapor pressure providing their superior safety profiles in comparison to the usual pressure‐liquified gases.^[^ ^28^, ^29^, ^30^ ^]^ In addition, it was found that both reactive ILs can be used as promising chlorination or hydrochlorination reagents, respectively.^[^ ^30^ ^]^

In this work, we investigated the performance and active site structure of a Pd catalyst for the hydrochlorination of acetylene to produce vinyl chloride when shifted from the gas to the liquid phase using the bichloride‐based IL [NEt_3_Me][Cl(HCl)*_n_*]. Taking inspiration from active and stable heterogeneous systems, we explored their homogeneous counterparts PtCl_2_ and AuCl in the bichloride. With this, we combine the ability of ILs to stabilize and influence catalytic reactions with the exceptional safety profile and inherent reactivity of the bichloride to enable a safer and easier production of VCM.

## Results and Discussion

At the outset, we investigated the hydrochlorination of acetylene using PdCl_2_ as a pre‐catalyst in the bichloride‐based ionic liquid [NEt_3_Me][Cl(HCl)*_n_*]. In a closed, pressure‐resistant flask, PdCl_2_ was mixed with [NEt_3_Me]Cl and then charged with HCl (1.2 equiv) and acetylene (1.0 equiv) forming the bichloride‐based IL [NEt_3_Me][Cl(HCl)_2.5_] in situ (**Table** **1**).

**Table 1:** General reaction equation (top) and yields for the hydrochlorination of acetylene using MCl_x_ as pre‐catalyst and the bichloride‐based ionic liquid [NEt_3_Me][Cl(HCl)_2.5_] (*T* = reaction temperature, *t* = reaction time). Isolated yields are given.

Reaction Conditions and Screening					
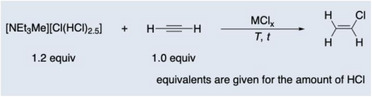					
Entry	MCl_x_	Cat. Loading [mol%]	*T* [°C]	*t* [h]	Yield [%]
1	PdCl_2_	0.1	100	12	50
2	PdCl_2_	0.5	100	12	99
3	PdCl_2_	1	100	12	99
4	PdCl_2_	0.5	20	16	15
5	PdCl_2_	0.5	50	16	31
6	PdCl_2_	0.5	150	12	Inseparable mixture
7	PtCl_2_	0.5	100	12	92
8	AuCl	0.5	100	12	7
9^a)^	PdCl_2_	0.5	100	12	< 2
10	–	–	100	20	10
11	[NEt_3_Me]_2_[PdCl_4_]	0.5	100	12	90
12^a)^	[NEt_3_Me]_2_[PdCl_4_]	0.5	100	20	16

^a)^ Use of HCl_(g)_ instead of [NEt_3_Me][Cl(HCl)_2.5_].

At 100 °C, a yield of 50% was observed for a catalyst loading of 0.1 mol% (**entry 1**), while in the presence of 0.5 mol% and 1.0 mol% PdCl_2_, VCM was isolated in yields of 99% (**entries 2** and **3**). When using temperatures of 20 and 50 °C, the yield dropped significantly, while a temperature of 150 °C resulted in the formation of an inseparable mixture of side products (**entries 4–6**).

While both PdCl_2_ and PtCl_2_ showed yields up to 99% (**entries 2** and **7**), using AuCl under comparable reaction conditions resulted in a substantially lower amount of product (7%, **entry 8**).

**Figure 1:**
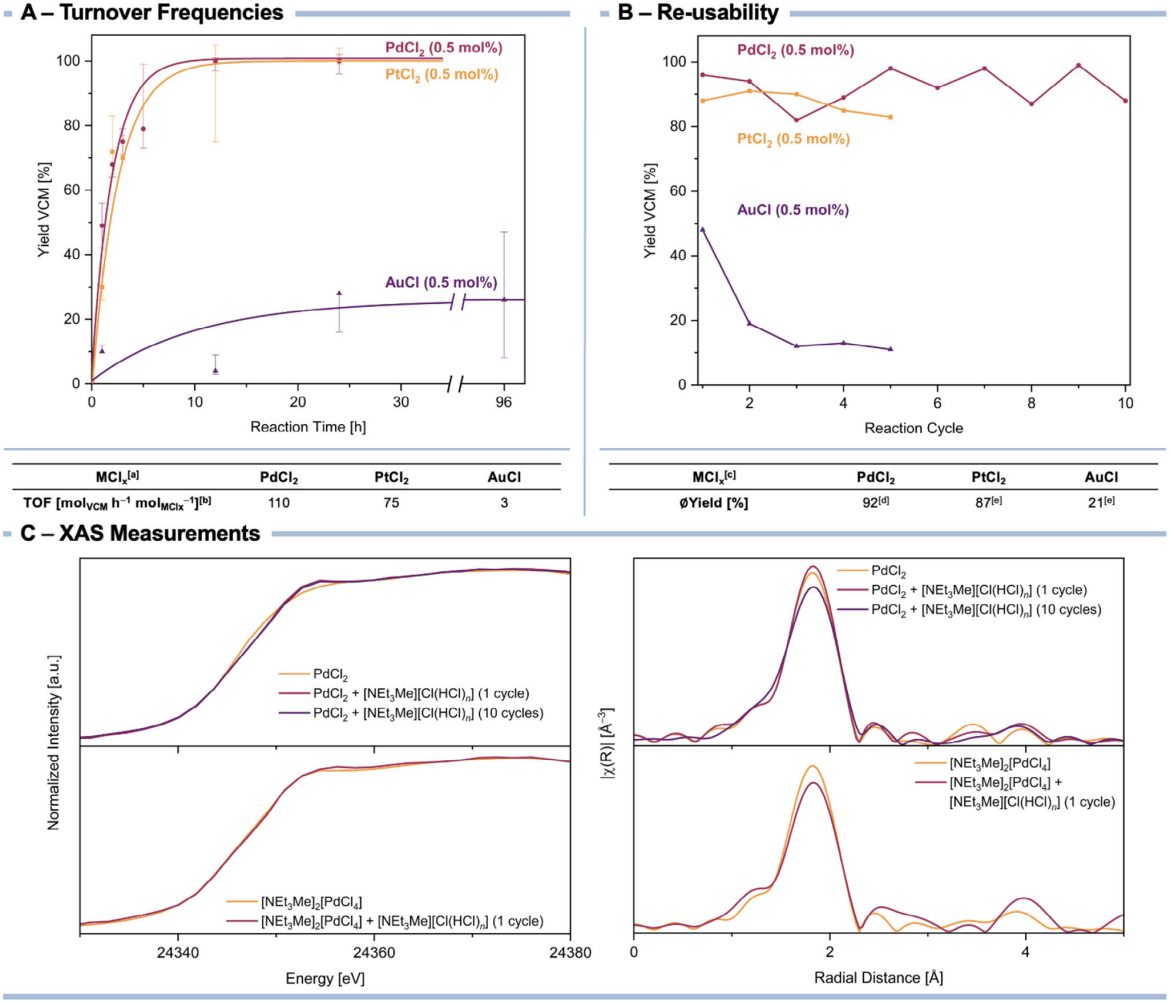
A) Yield of vinyl chloride (VCM) versus reaction time for the hydrochlorination of acetylene using the bichloride‐based ionic liquid [NEt_3_Me][Cl(HCl)_2.5_] and PdCl_2_ (●, red), PtCl_2_ (█, orange), or AuCl (▲, purple) as pre‐catalysts (top) and the resulting turnover frequencies (TOF) at yields of 20% (bottom). B) Yield of vinyl chloride (VCM) versus number of reaction cycles for the hydrochlorination of acetylene using the bichloride‐based ionic liquid [NEt_3_Me][Cl(HCl)_2.5_] and PdCl_2_ (●, red), PtCl_2_ (█, orange), or AuCl (▲, purple) as pre‐catalysts (top) and the resulting yields averaged over all reaction cycles (bottom). C) Pd *K* edge XANES (left) and EXAFS (right) spectra of PdCl_2_ and [NEt_3_Me]_2_[PdCl_4_] catalysts in fresh and used forms. ^[a]^Reactions were performed with a catalyst loading of 0.5 mol% and at a reaction temperature of 100 °C; ^[b]^values at a yield of 20%; ^[c]^reactions were performed with a catalyst loading of 0.5 mol%, at a reaction temperature of 100 °C, and over a reaction time of 20 h; ^[d]^average yield over ten reaction cycles; ^[e]^average yield over five reaction cycles.

To examine the difference of catalytic activities using PdCl_2_, PtCl_2_, and AuCl with [NEt_3_Me][Cl(HCl)*_n_*] in more detail, we analyzed the turnover frequency (TOF) of these systems at a yield of 20% (**Figure** **1**A; Figures S1–S3, Supporting Information). The TOF was obtained by determining the yields of VCM after different reaction times in triplicate.

A higher reaction rate was observed for PdCl_2_ (TOF = 110 mol_VCM_ h^−1^ mol_PdCl2_^−1^) compared to PtCl_2_ (TOF = 75 mol_VCM_ h^−1^ mol_PtCl2_^−1^).

PdCl_2_ showed a better initial catalytic activity providing a yield of 49% after 1 h, whereas PtCl_2_ produced VCM in a yield of 31% within the same time. Remarkably, the TOF of the PdCl_2_‐bichloride system exceeds the values of the gas phase process heterogeneously catalyzed by Pd proving its high efficiency and its applicability as a serious alternative to established catalysts.^[^ ^30^ ^]^ In contrast, using AuCl resulted in inconsistent yields and a much lower TOF of 3 mol_VCM_ h^−1^ mol_AuCl_^−1^.

In addition, we observed the dissolution of PdCl_2_ and PtCl_2_ in the bichloride indicating a homogeneous process which would require a reliable solubility of all reactants to facilitate the reaction. Our previous study showed that the bichloride stores up to 360 g L^−1^ of HCl (for [NEt_3_Me][Cl(HCl)_2.5_], 20 °C, 1 atm) providing a highly concentrated HCl environment.^[^ ^30^ ^]^ We determined the solubility of acetylene in the bichloride to be 3.9 g L^−1^ (0.1507 mol L^−1^) at 20 °C which is in accordance with values for other ILs (see Supporting Information).^[^ ^19^, ^22^, ^31^ ^]^ This indicates that the bichloride brings all reactants together in a liquid environment shifting the conversion of acetylene to VCM away from the gas phase which is further supported by the fact that in the absence of the bichloride, only traces of VCM could be isolated (Table 1, **entry 9**). The requirement of PdCl_2_ as a catalyst was proven by using the bichloride without a metal chloride resulting in a yield of only 10% (Table 1, **entry 10**).^[^ ^17^, ^18^, ^19^, ^20^, ^21^, ^22^, ^30^ ^]^

Additionally, vapor pressure investigations showed that the bichloride lowers the HCl pressure from 160 to 6.5 bar at 100 °C.^[^ ^30^ ^]^ This, the solubility of acetylene in the IL, and the occurring formation of VCM result in a significant decrease of the reaction pressure allowing us to perform the reactions in glassware without the need of high pressure equipment increasing the safety of VCM production in contrast to traditional acetylene hydrochlorination when just considering the dangers based on the high vapor pressure of HCl.^[^ ^32^ ^]^

**Figure 2:**
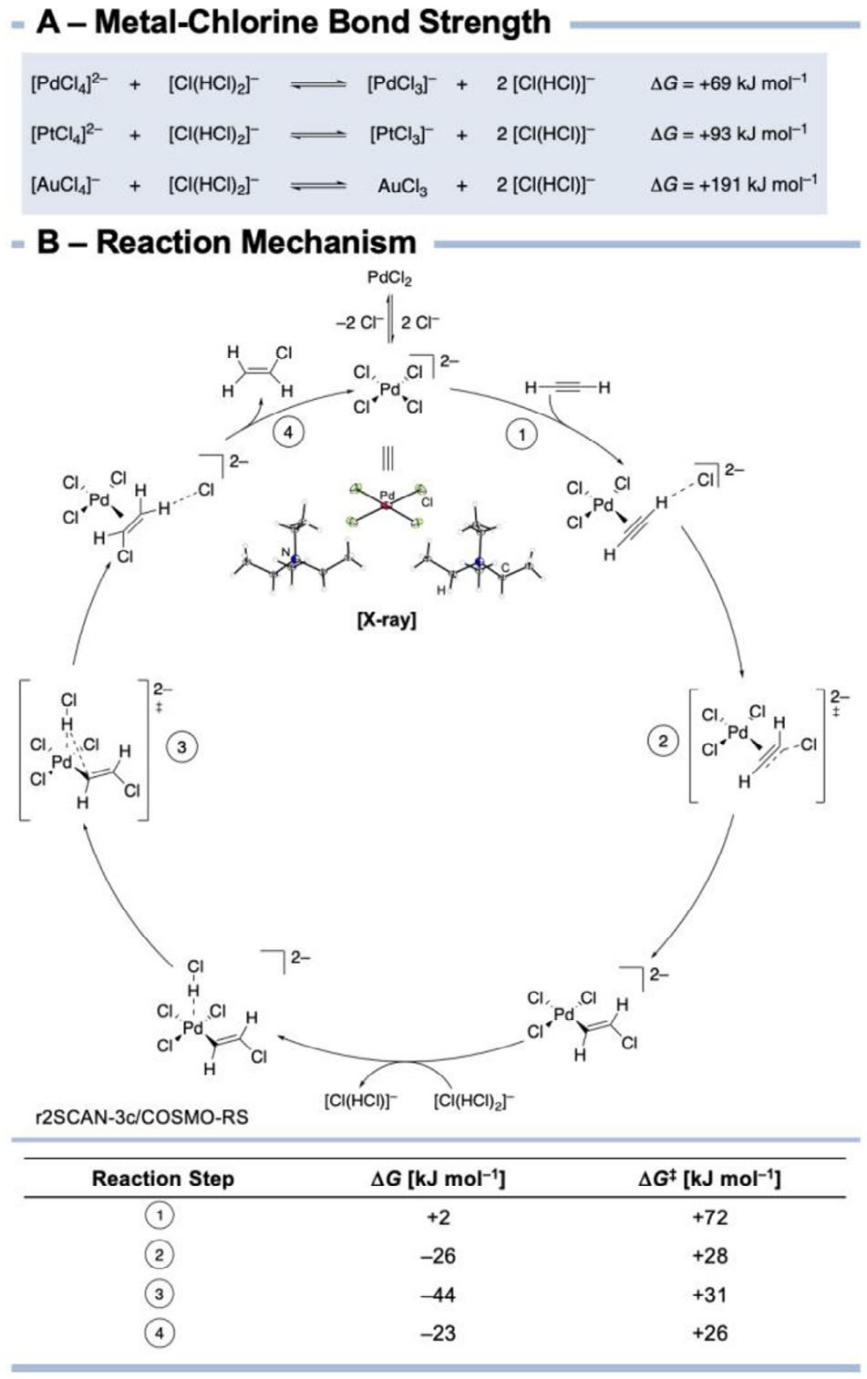
A) Free energies for the reactions of [MCl_4_]^x−^with the bichloride to estimate the metal‐chlorine bond strengths. B) Calculated reaction mechanism at r2SCAN‐3c/COSMO‐RS level of theory and molecular structure in the solid state of bis(triethylmethylammonium) tetrachloropalladate(ii) (with thermal ellipsoids are set at 50% probability). Reaction and activation free energies are given for each individual step.

When performing the hydrochlorination of acetylene in [NEt_3_Me][Cl(HCl)*_n_*] using AuCl as a catalyst, we noticed the formation of large, insoluble particles. Later, we observed that AuCl already reacts when mixed with [NEt_3_Me]Cl resulting in a color change from yellow to dark green. Powder diffraction measurements (see Figures S4,S8, and S9, Supporting Information) before and after adding HCl to [NEt_3_Me]Cl and AuCl revealed the presence of Au(0) in both cases. Based on the literature on the instability of Au(i), the disproportionation of AuCl to Au(0) and AuCl_3_ and further reaction to [AuCl_4_]^−^ is assumed to occur.^[^ ^33^, ^34^ ^]^ Since the observed particles are Au(0) and cannot be dissolved in [NEt_3_Me][Cl(HCl)*_n_*], we assume that a potential absence of interaction between bichloride and catalytically active gold species could be responsible for the slow hydrochlorination of acetylene. In addition, we performed quantum‐chemical calculations showing that the formed [AuCl_4_]^−^ is catalytically less active in the IL environment. This is due to a much more stabilized Au─Cl bond (191 kJ mol^−1^) when looking at the free energy of bichloride binding compared with the Pd─Cl bond in [PdCl_4_]^2−^ (69 kJ mol^−1^) and the Pt─Cl bond in [PtCl_4_]^2−^ (93 kJ mol^−1^) (**Figure** **2**A). Looking at acetylene binding energies, Pd and Au give similar values with −89.1 and −98.6 kJ mol^−1^, respectively, while Pt shows a stronger binding with −139.6 kJ mol^−1^. This may explain why Pd and Pt react similarly, but the TOF of PtCl_2_ is lower at a 20% yield of VCM.

Even more relevant for the potential applicability of the MCl_x_ bichloride system than its initial activity is the dynamic behavior of the active site and its catalytic robustness. Hence, we investigated the re‐usability of all three metal chlorides in combination with the bichloride (see Supporting Information). For this, we re‐loaded the system after a reaction cycle and re‐performed the reaction under the same conditions (*T* = 100 °C, *t* = 20 h, Figure 1B).

With an average yield of 92% compared to 87% with PtCl_2_ and 21% with AuCl, PdCl_2_ showed the best long‐term stability as a pre‐catalyst. The difference to PtCl_2_ is relatively low and might be a result of the yield fluctuations which is encouraged to be examined in greater depth in further studies. The activity of AuCl, however, drastically drops resulting in a constant yield of 10% after three reaction cycles corresponding to the yield when using the bichloride without metal chloride (Table 1, **entry 10**) showing the fast deactivation of the Au system (see above).

As we noted the dissolution of PdCl_2_ in the bichloride indicating a homogeneous process, we further analyzed the interaction between the Pd catalyst and the IL. Interestingly, when we slowly cooled a solution of PdCl_2_ and [NEt_3_Me][Cl(HCl)*_n_*] to −80 °C, we obtained red crystals suitable for X‐ray diffraction proving the formation of bis(triethylmethylammonium) tetrachloropalladate(II) ([NEt_3_Me]_2_[PdCl_4_]).^[^ ^35^ ^]^ Using [NEt_3_Me]_2_[PdCl_4_] directly as a catalyst resulted in the conversion of acetylene to VCM in a yield of 90% (Table 1, **entry 11**). Based on these results, we propose [NEt_3_Me]_2_[PdCl_4_] to be the catalytically active species, showing a remarkably high long‐term stability against deactivation and a high re‐usability. Interestingly, the combination of bichloride and [NEt_3_Me]_2_[PdCl_4_] seems to be necessary, since only 16% VCM were observed with gaseous HCl (Table 1, **entry 12**).

Further insights into the oxidation and coordination states of the Pd‐based catalyst were gained by X‐ray Absorption Spectroscopy (XAS) analyses (Figure 1C). Both PdCl_2_ and [NEt_3_Me]_2_[PdCl_4_] exhibit X‐ray Absorption Near Edge Structure (XANES) spectra in line with chlorinated Pd(ii) species. Upon use in acetylene hydrochlorination, the XANES spectral features of PdCl_2_ slightly change and more closely match those of [NEt_3_Me]_2_[PdCl_4_], further corroborating that the latter is the active species. More detailed information on the metal coordination environment was obtained by Extended X‐ray Absorption Fine Structure (EXAFS) analysis. First, no Pd─Pd bonding was detected in either PdCl_2_ or [NEt_3_Me]_2_[PdCl_4_] systems, highlighting the isolated nature of the active cationic metal centers. This was remarkably preserved after their use in acetylene hydrochlorination and is linked to the resistance of chlorinated Pd against being reduced by acetylene in the presence of the bichloride, which would lead to its deactivation as in the case of AuCl. In line with this, both systems, PdCl_2_ and [NEt_3_Me]_2_[PdCl_4_], exhibited a high chlorination degree of the Pd centers (Figure 1C, Pd─Cl coordination number, CN = 4.6 and 4.5, respectively), which is preserved after their use in a first reaction cycle (Figure 1C, Pd─Cl coordination number, CN = 4.8 and 4.1, respectively). Notably, EXAFS analysis shows no detectable Pd─C interactions, indicating the virtual absence of Pd–acetylene or Pd–coke species which can be reasoned by the highly concentrated HCl environment of the bichloride and the resulting high chlorination degree of the active Pd species. After ten reaction cycles, the PdCl_2_ system showed a minor contribution at 2.5 ± 0.03 Å that may be attributed to long bonding Pd–acetylene interactions (see Table S1, Supporting Information, Pd─C CN = 0.6).^[^ ^36^ ^]^ EXAFS analysis together with the stable performance of this catalytic system indicates that the active site is a tetrachlorinated Pd center with a good resistance to metal reduction and coke formation.

To gain a better understanding of the process and the system stability, we performed quantum‐chemical calculations at the r^2^SCAN‐3c^[^ ^37^ ^]^ level to elucidate the reaction mechanism (Figure 2B).

Starting from [PdCl_4_]^2−^, we found the introduction of acetylene to the coordination sphere of the palladium center to be the rate determining step (step 1). The moderate barrier of 72 kJ mol^−1^ matches well with the relatively high experimental yields at moderate temperatures. Upon coordination of acetylene, a chloride ion forms the C─Cl bond (step 2), resulting in a Pd─C σ bond. This bond is then protonated by an external HCl molecule (step 3) forming the product. Upon dissociation of vinyl chloride, the active catalyst is reformed. The barriers of the last three steps are significantly lower than those of the rate determining step 1.

However, comparative computations with Pt instead of Pd in step 1 give a 24 kJ mol^−1^ higher barrier consistent with the experimental findings discussed above. Au gives a very similar barrier as Pt (+19 kJ mol^−1^ higher than Pd). However, the initial step is almost 100 kJ mol^−1^ endergonic, so the reaction will most likely not proceed. This can be traced back to the significantly larger Au─Cl binding energy (Figure 2A).

While we estimate the effects of the IL via the COSMO‐RS solvent model^[^ ^38^, ^39^, ^40^ ^]^ we suspect that explicit hydrogen bonding interaction with HCl from the solvent or chloride exchange reactions might lower the barrier even further. However, the explicit consideration of an additional HCl molecule in the mechanism caused numerical instabilities resulting from the surrounding COSMO solvation model.^[^ ^41^ ^]^ Interestingly, the comparatively low barrier heights found in our calculations do not indicate a specific requirement for the bichloride ion to be present during the reaction. We therefore conclude that the IL most likely enables very high local concentrations of HCl and the stabilization of chloride ions formed during the reaction. We explicitly note, that the bichloride ion does not coordinate to the Pd‐center directly but splits into a chloride ion and HCl. Obviously, we cannot exclude a scrambling between chloride ions coordinating weakly to the catalytic species and those present within the IL.

## Conclusion

In conclusion, the combination of the bichloride‐based ionic liquid [NEt_3_Me][Cl(HCl)*_n_*] with PdCl_2_ results in the formation of the active catalyst [NEt_3_Me]_2_[PdCl_4_] for the mercury‐free hydrochlorination of acetylene. Its highly chlorinated structure enables resistance to metal reduction and coking, yielding in a stable catalytic performance and re‐usability over ten reaction cycles (200 h in total) at TOF = 110 mol_VCM_ h^−1^ mol_PdCl2_^−1^, in a similar VCM productivity ballpark as heterogeneous noble metal catalysts for gas‐phase acetylene hydrochlorination.^[^ ^29^ ^]^ Replacing gaseous HCl with the bichloride‐based IL shifts the whole process into the liquid phase decreasing the reaction pressure to offer a safer route for VCM production regarding the handling of HCl. This means that other materials than, e.g., steel could be used to reduce production costs and corrosion problems. Preliminary catalytic investigations reported in this study show promising results for wide applicability of the bichloride‐based IL technology to other metals, such as platinum, and underline the large possibilities of reactive ILs for potential industrially relevant processes.

## Conflict of Interest

The authors declare no conflict of interest.

## Supporting information



Supporting Information

## Data Availability

The data that support the findings of this study are available from the corresponding author upon reasonable request.
