# Supplementary material for: Shifting Acetylene Hydrochlorination From the Gas to the Liquid Phase: Vinyl Chloride Production in Bichloride‐Based Ionic Liquids
Source: Adv Sci (Weinh). 2025 Jun 10;12(32):e03992. doi: 10.1002/advs.202503992 (PMC12407274; doi:10.1002/advs.202503992)
Supplement: Supplementary file 1 — Supporting Information [file ADVS-12-e03992-s001.pdf]

ADVANCED SCIENCE
Open Access

Supporting Information

for *Adv. Sci.*, DOI 10.1002/advs.202503992

Shifting Acetylene Hydrochlorination From the Gas to the Liquid Phase: Vinyl Chloride Production in Bichloride-Based Ionic Liquids

Gesa H. Dreyhsig, Vera Giulimondi, Merlin Kleoff, Letizia Lanfredi, Maxim Fuchs, Niklas Limberg, Marc Reimann, Martin Kaupp, Javier Pérez-Ramírez and Sebastian Riedel**

Supporting Information for

Shifting Acetylene Hydrochlorination from the Gas to the Liquid Phase: Vinyl Chloride Production in Bichloride-based Ionic Liquids

Gesa H. Dreyhsig,[a] Vera Giulimondi,[b] Merlin Kleoff,[a] Letizia Lanfredi,[a] Maxim Fuchs,[a] Niklas Limberg,[a] Marc Reimann,[c] Martin Kaupp,[c] Javier Pérez-Ramírez,[b]* Sebastian Riedel[a]*

Corresponding authors: s.riedel@fu-berlin.de, jpr@chem.ethz.ch

The PDF file includes:

Materials and Methods
Schemes S1 to S11
Figures S1 to S10
Tables S1 to S2
References

Table of contents

Materials and methods…… 3

General procedures for the preparation of [NEt_3Me][$Cl(HCl)_n$]…… 3

Synthesis of vinyl chloride using [NEt_3Me][$Cl(HCl)_n$] and $PdCl_2$…… 4

Synthesis of vinyl chloride using [NEt_3Me][$Cl(HCl)_n$] and $PtCl_2$…… 5

Synthesis of vinyl chloride using [NEt_3Me][$Cl(HCl)_n$] and AuCl…… 6

Synthesis of [NEt_3Me]$_2$[$PdCl_4$] …… 6

Control Experiments…… 7

Determination of the turnover frequency…… 10

Investigation of the long-term stability and re-usability of the bichloride-catalyst system…… 12

Characterization of the decomposition of AuCl…… 13

Solubility of acetylene in [NEt_3Me][$Cl(HCl)_{2.5}$]…… 14

Catalyst characterization…… 14

IR Spectra…… 15

Powder Diffractometry…… 17

Molecular structures in the solid state…… 18

Crystal data…… 19

Computational details…… 20

Cartesian coordinates…… 20

References…… 26

Materials and methods

Attention: all reactions were performed using gaseous hydrogen chloride and acetylene, use appropriate safety precautions and handle with care.

All substances sensitive to water and oxygen were handled under an argon atmosphere using standard Schlenk techniques and an oil pump vacuum up to 10^{-3} mbar. All chemicals were obtained from commercial suppliers and were used without further purification if not stated otherwise. $[NEt_3Me]Cl$ was dried in vacuo at 100 °C for 1 hour to 1 day prior to use, while all solvents were obtained anhydrous by storage over activated 3 Å molecular sieves. All glassware was washed carefully several times with aqua regia to avoid any metal residues from previous experiments. X-ray diffraction data were collected on a Bruker D8 Venture CMOS area detector (Photon 100) diffractometer with $Mo_{K\alpha}$ radiation. Single crystals and powders were coated with perfluoroether oil at low temperatures around –80 °C and mounted on a 0.1–0.2 mm Micromount. The structures were solved with the ShelXT structure solution program using intrinsic phasing and refined with the ShelXT refinement package using least squares on weighted F2 values for all reflections using OLEX2.[1-3] Hydrogen atoms were treated using the HFIX 23 (CH_2 groups) and HFIX 137 (CH_3 groups) restrains as implemented in ShelXL. Powder X-ray diffractograms were measured using $Cu_{K\alpha}$ radiation via a 360° Phi scan with an exposure time of 120 s. The data were integrated using APEX5 and analyzed using DIFFRAC.EVA. IR spectra were recorded using a Nicolet™ iS50 FT-IR spectrometer. OPUS 7.5 and OMNIC were used for evaluation of the recorded spectra, Origin 2022 for the graphical representation.

General procedure for the preparation of $[NEt_3Me][Cl(HCl)_n]$

The bichloride $[NEt_3Me][Cl(HCl)_n]$ was prepared according to a reported procedure with a defined stoichiometry by condensing the calculated amount of HCl onto dry triethylmethylammonium chloride $[NEt_3Me]Cl$ and was obtained as a colorless, viscous liquid.[4]

Disposal of the bichloride

If necessary, the bichloride can be diluted with water to obtain hydrochloric acid and can be discarded after careful neutralization with sodium bicarbonate.

Synthesis of vinyl chloride using [NEt_3Me][Cl(HCl)$_n$] and $PdCl_2$

<u>Optimized reaction conditions</u>

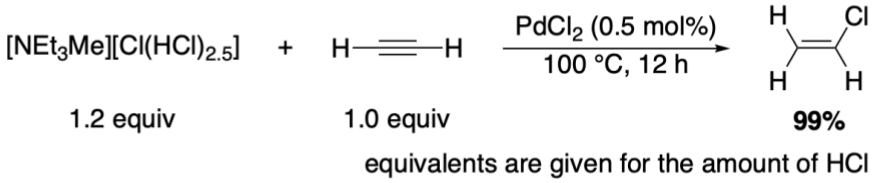

Scheme S1 Hydrochlorination of acetylene using the bichloride-based Ionic Liquid [NEt_3Me][Cl(HCl)$_{2.5}$] and $PdCl_2$ under optimized reaction conditions.

In a glovebox, [NEt_3Me]Cl (0.821 g, 5.41 mmol, 0.480 equiv) and $PdCl_2$ (10.0 mg, 0.0564 mmol, 0.5 mol%) were mixed and transferred to a 100 mL-Rettberg flask. HCl (0.494 g, 13.5 mmol, 1.20 equiv) was condensed onto the solids to form the bichloride [NEt_3Me][Cl(HCl)$_{2.5}$] (1.32 g, 5.41 mmol, 0.480 equiv). After warming to room temperature, the exact amount of HCl was determined gravimetrically and adjusted if necessary. Subsequently, acetylene (0.294 g, 11.3 mmol, 1.00 equiv) was condensed onto the frozen mixture, warmed to room temperature, the exact amount determined gravimetrically, and adjusted if necessary. The reaction mixture was heated to 100 °C in a pre-heated oil bath and stirred for 12 h. After cooling the reaction mixture to 0 °C, the products were obtained by distilling the volatile constituents in vacuo using cooling traps held at –138 °C (VCM) and –198 °C (HCl, C_2H_2). All components were identified by their gas-phase IR spectra (**Figure S5 and S6**). The VCM fraction was transferred into a pressure-resistant Rettberg flask to determine the mass of VCM gravimetrically (0.699 g, 11.2 mmol, **99%**). The spectroscopic data are consistent with those in the literature.[5]

<u>Screening conditions</u>

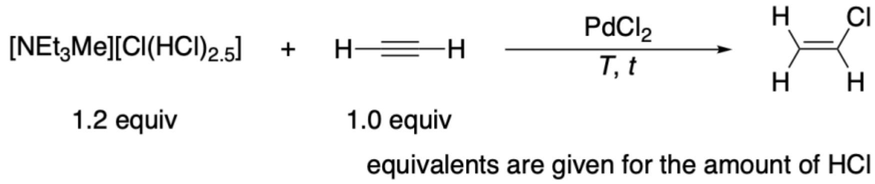

Scheme S2 Hydrochlorination of acetylene using the bichloride-based Ionic Liquid [NEt_3Me][Cl(HCl)$_{2.5}$] and $PdCl_2$ under screening conditions (T = reaction temperature, t = reaction time).

In a glovebox, [NEt_3Me]Cl (0.480 equiv) and $PdCl_2$ were mixed and transferred to a 100 mL-Rettberg flask. HCl (1.20 equiv) was condensed onto the solids to form the bichloride [NEt_3Me][Cl(HCl)$_{2.5}$] (0.480 equiv). After warming to room temperature, the exact amount of HCl was determined gravimetrically and adjusted if necessary. Subsequently, acetylene (1.00 equiv)

was condensed onto the frozen mixture, warmed to room temperature, the exact amount determined gravimetrically, and adjusted if necessary. The reaction mixture was heated to the desired temperature *T* in a pre-heated oil bath and stirred for the intended time *t*. After cooling the reaction mixture to 0 °C, the products were obtained by distilling the volatile constituents in vacuo using cooling traps held at –138 °C (VCM) and –198 °C (HCl, C_2H_2). All components were identified by their gas-phase IR spectra (**Figure S5 and S6**). The VCM fraction was transferred into a pressure-resistant Rettberg flask to determine the mass of VCM gravimetrically. The spectroscopic data are consistent with those in the literature.[5]
See also Table 1 in the manuscript.

Synthesis of vinyl chloride using $[NEt_3Me][Cl(HCl)_n]$ and $PtCl_2$

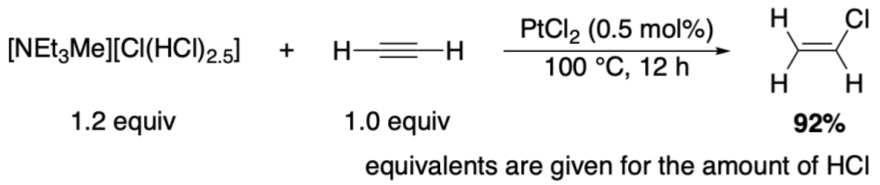

Scheme S3 Hydrochlorination of acetylene using the bichloride-based Ionic Liquid $[NEt_3Me][Cl(HCl)_{2.5}]$ and $PtCl_2$.

In a glovebox, $[NEt_3Me]Cl$ (0.547 g, 3.61 mmol, 0.480 equiv) and $PtCl_2$ (10.0 mg, 0.0376 mmol, 0.5 mol%) were mixed and transferred to a 100 mL-Rettberg flask. HCl (0.329 g, 9.02 mmol, 1.20 equiv). was condensed onto the solids to form the bichloride $[NEt_3Me][Cl(HCl)_{2.5}]$ (0.876 g, 3.61 mmol, 0.480 equiv). After warming to room temperature, the exact amount of HCl was determined gravimetrically and adjusted if necessary. Subsequently, acetylene (0.196 g, 7.52 mmol, 1.00 equiv) was condensed onto the frozen mixture, warmed to room temperature, the exact amount determined gravimetrically, and adjusted if necessary. The reaction mixture was heated to 100 °C in a pre-heated oil bath and stirred for 12 h. After cooling the reaction mixture to 0 °C, the products were obtained by distilling the volatile constituents in vacuo using cooling traps held at –138 °C (VCM) and –198 °C (HCl, C_2H_2). All components were identified by their gas-phase IR spectra (**Figure S5 and S6**). The VCM fraction was transferred into a pressure-resistant Rettberg flask to determine the mass of VCM gravimetrically (0.432 g, 6.92 mmol, **92%**). The spectroscopic data are consistent with those in the literature.[5]

Synthesis of vinyl chloride using [NEt_3Me][Cl(HCl)$_n$] and AuCl

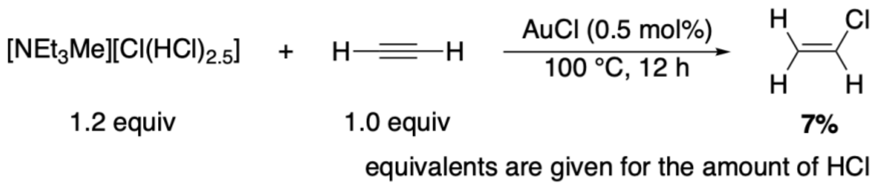

Scheme S4 Hydrochlorination of acetylene using the bichloride-based Ionic Liquid [NEt_3Me][$Cl(HCl)_{2.5}$] and AuCl.

In a glovebox, [NEt_3Me]Cl (0.626 g, 4.13 mmol, 0.480 equiv) and AuCl (10.0 mg, 0.0376 mmol, 0.5 mol%) were mixed and transferred to a 100 mL-Rettberg flask. HCl (0.377 g, 10.3 mmol, 1.20 equiv) was condensed onto the solids to form the bichloride [NEt_3Me][$Cl(HCl)_{2.5}$] (1.00 g, 4.13 mmol, 0.480 equiv). After warming to room temperature, the exact amount of HCl was determined gravimetrically and adjusted if necessary. Subsequently, acetylene (0.224 g, 8.61 mmol, 1.00 equiv) was condensed onto the frozen mixture, warmed to room temperature, the exact amount determined gravimetrically, and adjusted if necessary. The reaction mixture was heated to 100 °C in a pre-heated oil bath and stirred for 12 h. After cooling the reaction mixture to 0 °C, the products were obtained by distilling the volatile constituents in vacuo using cooling traps held at –138 °C (VCM) and –198 °C (HCl, C_2H_2). All components were identified by their gas-phase IR spectra (**Figure S5 and S6**). The VCM fraction was transferred into a pressure-resistant Rettberg flask to determine the mass of VCM gravimetrically (0.0377 g, 0.603 mmol, **7%**). The spectroscopic data are consistent with those in the literature.[5]

Synthesis of [NEt_3Me]$_2$[$PdCl_4$]

<u>Crystal formation</u>

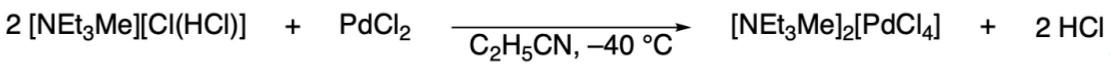

Scheme S5 Synthesis of [NEt_3Me]$_2$[$PdCl_4$] by the reaction of the bichloride-based Ionic Liquid [NEt_3Me][Cl(HCl)] and $PdCl_2$ in propionitrile.

In a glovebox, [NEt_3Me]Cl (8.60 mg, 0.0567 mmol, 1.00 equiv) and $PdCl_2$ (5.00 mg, 0.0282 mmol, 2.01 equiv) were mixed in a 6.5 mL-Rettberg tube and dissolved in propionitrile (1.50 mL). Subsequently, HCl (2.07mg, 0.0567 mmol, 1.00 equiv) was condensed onto the frozen mixture before slowly warming to room temperature to check for the complete dissolution of all components. Red crystals of [NEt_3Me]$_2$[$PdCl_4$] suitable for X-ray diffraction were obtained by slowly cooling the solution to –80 °C (**Figure S10**, **Table S2**).

Synthesis of catalyst for control experiments

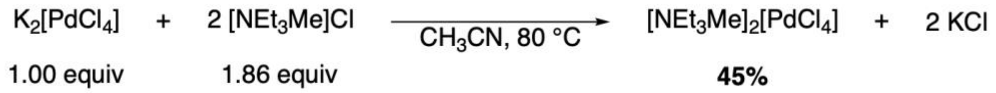

Scheme S6 Synthesis of $[NEt_3Me]_2[PdCl_4]$ by the reaction of $K_2[PdCl_4]$and $[NEt_3Me]Cl$ in acetonitrile.

$K_2[PdCl_4]$ (1.50 g, 4.60 mmol, 1.00 equiv) was mixed with $[NEt_3Me]Cl$ (1.30 g, 8.57 mmol, 1.86 equiv) and dissolved in acetonitrile (35.0 mL). The reaction mixture was stirred under reflux, whereby the solution turned red and a colorless solid precipitated. After three hours, the still warm reaction mixture was filtered, washed with warm acetonitrile (3x 10 mL) and dried in vacuo at 45 °C. $[NEt_3Me]_2[PdCl_4]$ was obtained as a red powder (1.00 g, 2.08 mmol, **45%**, **Figure S7**).

$[NEt_3Me]_2[PdCl_4]$: IR (ATR), $\tilde{\nu}$ = 2983, 1484, 1444, 1402, 1371, 1348, 1317, 1284, 1204, 1191, 1127, 1075, 1009, 955, 926, 873, 810, 792, 759, 536, 498, 441 cm^{-1}.

Control experiments

Using $[NEt_3Me][Cl(HCl)_n]$ without MCl_x

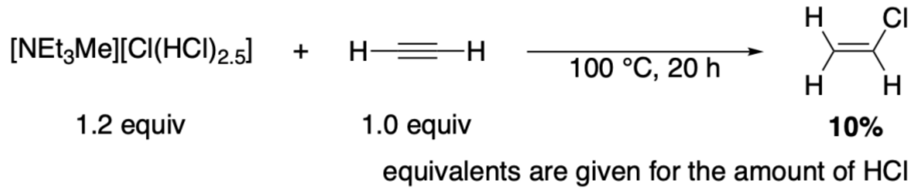

Scheme S7 Hydrochlorination of acetylene using the bichloride-based Ionic Liquid $[NEt_3Me][Cl(HCl)_{2.5}]$ without a metal based catalyst.

In a glovebox, $[NEt_3Me]Cl$ (0.303 g, 2.00 mmol, 0.480 equiv) was transferred to a 100 mL-Rettberg flask. HCl (0.182 g, 4.99 mmol, 1.20 equiv) was condensed onto the solids to form the bichloride $[NEt_3Me][Cl(HCl)_{2.5}]$ (0.411 g, 2.00 mmol, 0.480 equiv). After warming to room temperature, the exact amount of HCl was determined gravimetrically and adjusted if necessary. Subsequently, acetylene (0.108 g, 4.16 mmol, 1.00 equiv) was condensed onto the frozen mixture, warmed to room temperature, the exact amount determined gravimetrically, and adjusted if necessary. The reaction mixture was heated to 100 °C in a pre-heated oil bath and stirred for 20 h. After cooling the reaction mixture to 0 °C, the products were obtained by distilling the volatile constituents in vacuo using cooling traps held at –138 °C (VCM) and –198 °C (HCl, C_2H_2). All components were identified by their gas-phase IR spectra (**Figure S5 and S6**). The VCM fraction was transferred into a pressure-resistant Rettberg flask to determine the mass of VCM

gravimetrically (0.0260 g, 0.416 mmol, **10%**). The spectroscopic data are consistent with those in the literature.[5]

Using $HCl_{(g)}$ as hydrochlorination reagent

Attention: this reaction is under high pressure, use appropriate safety precautions.

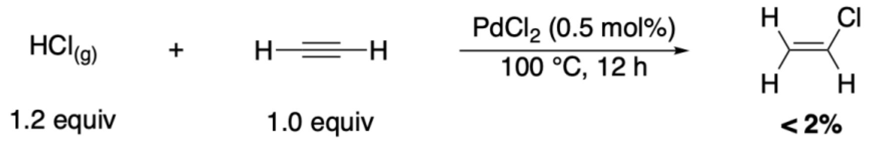

Scheme S8 Hydrochlorination of acetylene using the gaseous hydrogen chloride and $PdCl_2$

In a glovebox, $PdCl_2$ (10.0 mg, 0.0564 mmol, 0.5 mol%) was transferred to a 100 mL-Rettberg flask and HCl (0.494 g, 13.5 mmol, 1.20 equiv) was condensed onto the solid. After warming to room temperature, the exact amount of HCl was determined gravimetrically and adjusted if necessary. Subsequently, acetylene (0.294 g, 11.3 mmol, 1.00 equiv) was condensed onto the frozen mixture, warmed to room temperature, the exact amount determined gravimetrically, and adjusted if necessary. The reaction mixture was heated to 100 °C in a pre-heated oil bath and stirred for 12 h. After cooling the reaction mixture to 0 °C, the products were obtained by distilling the volatile constituents in vacuo using cooling traps held at –138 °C (VCM) and –198 °C (HCl, C_2H_2). All components were identified by their gas-phase IR spectra (**Figure S5 and S6**). The VCM fraction was transferred into a pressure-resistant Rettberg flask to determine the mass of VCM gravimetrically (**< 2%**). The spectroscopic data are consistent with those in the literature.[5]

Using $[NEt_3Me]_2[PdCl_4]$ as pre-catalyst

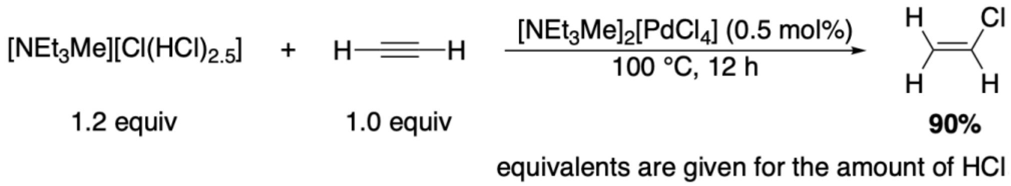

Scheme S9 Hydrochlorination of acetylene using the bichloride-based Ionic Liquid $[NEt_3Me][Cl(HCl)_{2.5}]$ and $[NEt_3Me]_2[PdCl_4]$.

In a glovebox, $[NEt_3Me]Cl$ (0.303 g, 2.00 mmol, 0.480 equiv) and $[NEt_3Me]_2[PdCl_4]$ (10.0 mg, 0.0208 mmol, 0.5 mol%) were mixed and transferred to a 100 mL-Rettberg flask. HCl (0.182 g, 4.99 mmol, 1.20 equiv) was condensed onto the solids to form the bichloride $[NEt_3Me][Cl(HCl)_{2.5}]$ (1.00 g, 4.12 mmol, 0.480 equiv). After warming to room temperature, the exact amount of HCl was determined gravimetrically and adjusted if necessary. Subsequently, acetylene (0.108 g,

4.16 mmol, 1.00 equiv) was condensed onto the frozen mixture, warmed to room temperature, the exact amount determined gravimetrically, and adjusted if necessary. The reaction mixture was heated to 100 °C in a pre-heated oil bath and stirred for 12 h. After cooling the reaction mixture to 0 °C, the products were obtained by distilling the volatile constituents in vacuo using cooling traps held at –138 °C (VCM) and –198 °C (HCl, C_2H_2). All components were identified by their gas-phase IR spectra (**Figure S5 and S6**). The VCM fraction was transferred into a pressure-resistant Rettberg flask to determine the mass of VCM gravimetrically (0.234 g, 3.74 mmol, **90%**). The spectroscopic data are consistent with those in the literature.[5]

Using $HCl_{(g)}$ as hydrochlorination reagent and $[NEt_3Me]_2[PdCl_4]$ as pre-catalyst

Attention: this reaction is under high pressure, use appropriate safety precautions.

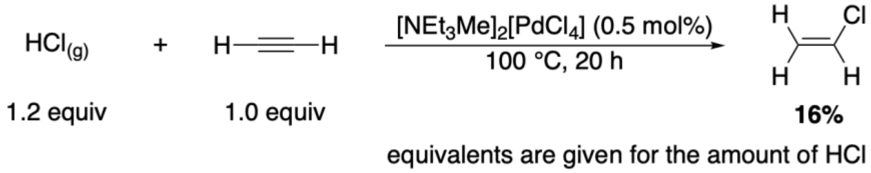

Scheme S10 Hydrochlorination of acetylene using the bichloride-based Ionic Liquid $[NEt_3Me][Cl(HCl)_{2.5}]$ and $[NEt_3Me]_2[PdCl_4]$.

In a glovebox, $[NEt_3Me]_2[PdCl_4]$ (10.0 mg, 0.0208 mmol, 0.5 mol%) was transferred to a 100 mL-Rettberg flask. HCl (0.182 g, 4.99 mmol, 1.20 equiv) was condensed onto the solid. After warming to room temperature, the exact amount of HCl was determined gravimetrically and adjusted if necessary. Subsequently, acetylene (0.108 g, 4.16 mmol, 1.00 equiv) was condensed onto the frozen mixture, warmed to room temperature, the exact amount determined gravimetrically, and adjusted if necessary. The reaction mixture was heated to 100 °C in a pre-heated oil bath and stirred for 20 h. After cooling the reaction mixture to 0 °C, the products were obtained by distilling the volatile constituents in vacuo using cooling traps held at –138 °C (VCM) and –198 °C (HCl, C_2H_2). All components were identified by their gas-phase IR spectra (**Figure S5 and S6**). The VCM fraction was transferred into a pressure-resistant Rettberg flask to determine the mass of VCM gravimetrically (0.0400 g, 0.640 mmol, **16%**). The spectroscopic data are consistent with those in the literature.[5]

Determination of the turnover frequency

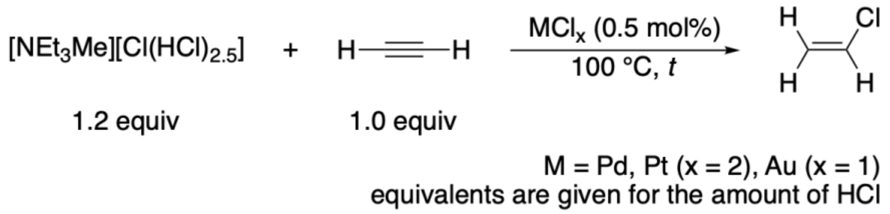

Scheme S11 Hydrochlorination of acetylene using the bichloride-based Ionic Liquid $[NEt_3Me][Cl(HCl)_{2.5}]$ and MCl_x (M = Pd, Pt with x = 2, M = Au with x = 1; *t* = reaction time).

Turnover frequencies (TOFs) were determined using isolated yields. All reactions were performed in triplicate to take into account the error of the method. The fits were obtained by using the analysis tool of Origin 2022 and the TOFs determined at a yield of 20% (**Figures S1-S3**).

In a glovebox, $[NEt_3Me]Cl$ (0.480 equiv) and MCl_x (0.5mol%) were mixed and transferred to a 100 mL-Rettberg flask. HCl (1.20 equiv) was condensed onto the solids to form the bichloride $[NEt_3Me][Cl(HCl)_{2.5}]$ (0.480 equiv). After warming to room temperature, the exact amount of HCl was determined gravimetrically and adjusted if necessary. Subsequently, acetylene (1.00 equiv) was condensed onto the frozen mixture, warmed to room temperature, the exact amount determined gravimetrically, and adjusted if necessary. The reaction mixture was heated to 100 °C in a pre-heated oil bath and stirred for the intended time *t*. After cooling the reaction mixture to 0 °C, the products were obtained by distilling the volatile constituents in vacuo using cooling traps held at –138 °C (VCM) and –198 °C (HCl, C_2H_2). All components were identified by their gas-phase IR spectra (**Figure S5 and S6**). The VCM fraction was transferred into a pressure-resistant Rettberg flask to determine the mass of VCM gravimetrically. The spectroscopic data are consistent with those in the literature.[5]

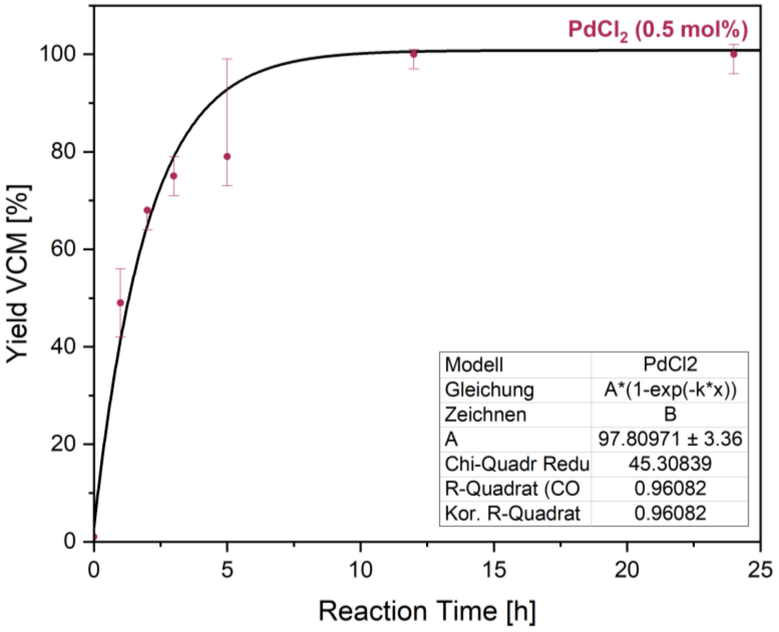

Figure S1 Yield of vinyl chloride versus time plot for the hydrochlorination of acetylene using the bichloride-based Ionic Liquid [NEt$_3$Me][Cl(HCl)$_{2.5}$] and $PdCl_2$ and the used fit function to obtain the turnover frequency. TOF at a yield of 20% = 110 h^{-1}.

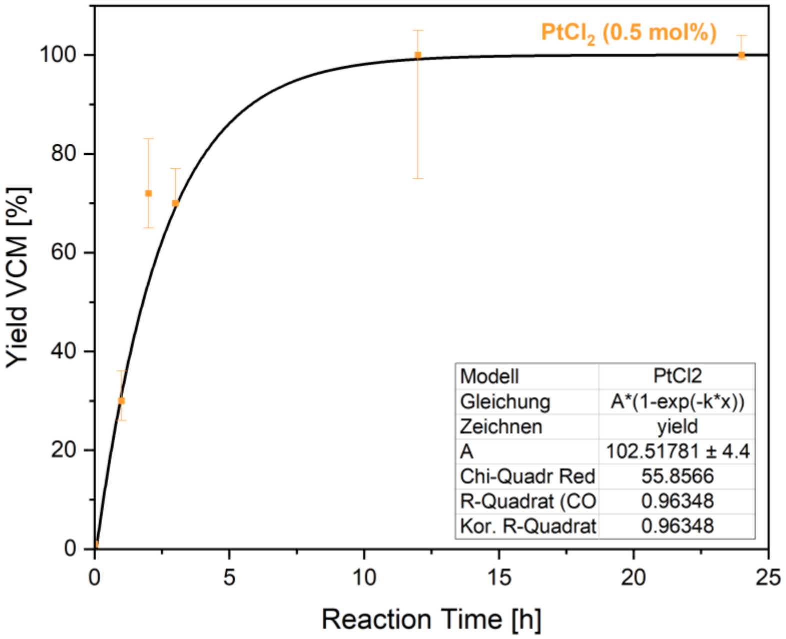

Figure S2 Yield of vinyl chloride versus time plot for the hydrochlorination of acetylene using the bichloride-based Ionic Liquid [NEt$_3$Me][Cl(HCl)$_{2.5}$] and $PtCl_2$ and the used fit function to obtain the turnover frequency. TOF at a yield of 20% = 75 h^{-1}.

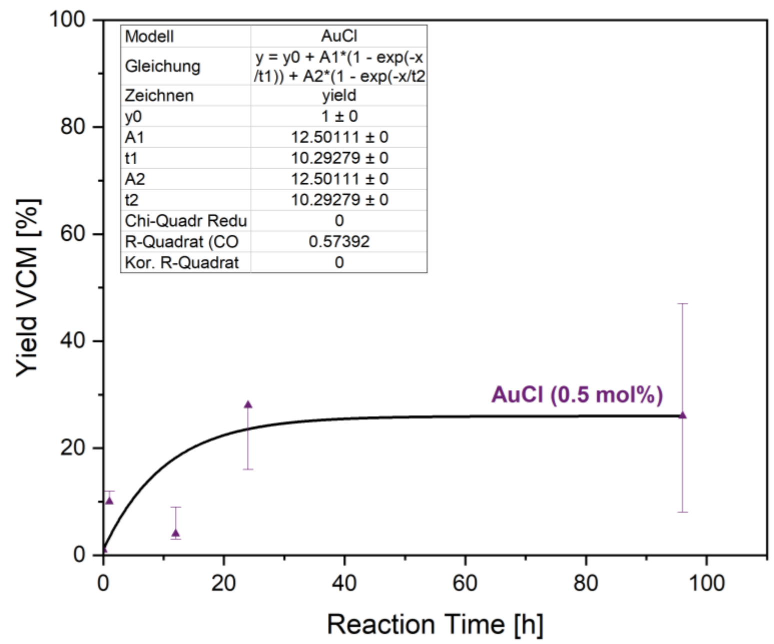

Figure S3 Yield of vinyl chloride versus time plot for the hydrochlorination of acetylene using the bichloride-based Ionic Liquid [NEt_3Me][Cl(HCl)$_{2.5}$] and AuCl and the used fit function to obtain the turnover frequency. TOF at a yield of 20% = 3 h^{-1}.

Investigation of the long-term stability and re-usability of the bichloride-catalyst system

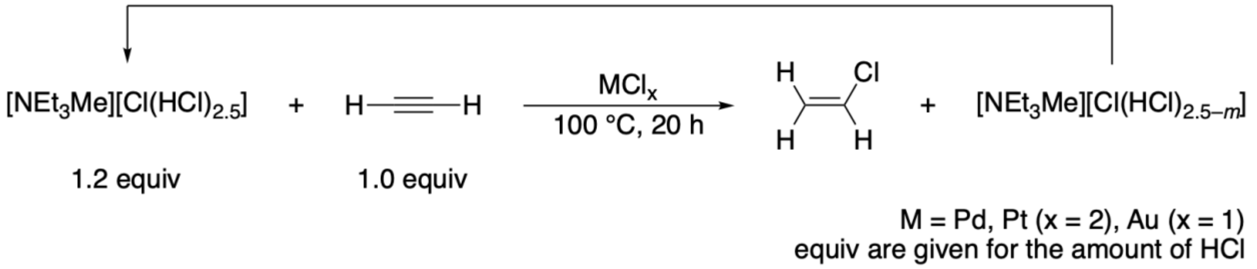

Scheme S12 Hydrochlorination of acetylene using the bichloride-based Ionic Liquid [NEt_3Me][Cl(HCl)$_{2.5}$] and MCl_x (M = Pd, Pt with x = 2, M = Au with x = 1) and re-loading the system with the consumed amount of HCl and acetylene.

All long-term stabilities of the bichloride-catalyst system were determined by re-loading the already used system.

In a glovebox, [NEt_3Me]Cl (0.480 equiv) and MCl_x (0.5 mol%) were mixed and transferred to a 100 mL-Rettberg flask. HCl (1.20 equiv) was condensed onto the solids to form the bichloride [NEt_3Me][Cl(HCl)$_{2.5}$] (0.480 equiv). After warming to room temperature, the exact amount of HCl was determined gravimetrically and adjusted if necessary. Subsequently, acetylene (1.00 equiv)

was condensed onto the frozen mixture, warmed to room temperature, the exact amount determined gravimetrically, and adjusted if necessary. The reaction mixture was heated to 100 °C in a pre-heated oil bath and stirred for 20 h. After cooling the reaction mixture to 0 °C, the products were obtained by distilling the volatile constituents in vacuo using cooling traps held at –138 °C (VCM) and –198 °C (HCl, C_2H_2). All components were identified by their gas-phase IR spectra (**Figure S5 and S6**). The VCM fraction was transferred into a pressure-resistant Rettberg flask to determine the mass of VCM gravimetrically. The spectroscopic data are consistent with those in the literature.[5]

Due to the strength of the present 3c-4e [Cl–HCl] bond in the bichloride anion,[4] even prolonged evacuation at high temperatures of the reaction residuum cannot remove all the HCl from the bichloride. That, in connection with performing the reaction with an excess of HCl, result in HCl still present in the reaction mixture after a reaction cycle. To proof this, we prepared colorless crystals suitable for X-ray diffractometry by dissolving the remaining residuum in acetonitrile and slowly cooling this mixture to –40 °C proving the presence of [NEt_3Me][Cl(HCl)].[4] We determined the quantity of left HCl gravimetrically and re-loaded the system with the deviation to regenerate the initial amount (1.20 equiv). Since no acetylene was left after the reaction or after evacuating the bichloride over a longer period, the system was directly loaded with the same amount of acetylene (1.00 equiv) after each reaction cycle.

Characterization of the decomposition of AuCl

We observed a rapid color change from pale yellow to dark green/black when bringing [NEt_3Me]Cl and AuCl in contact with each other under an argon atmosphere. To investigate this in more detail, we prepared a sample of [NEt_3Me]Cl and AuCl suitable for X-ray powder diffractometry to measure a XRPD pattern (**Figure S8**). The spectroscopic data are in agreement with the literature data of Au(0) and also $AuCl_3$ whereas no signals of AuCl can be found.[6-8] As a result, we assume the disproportion of AuCl to Au(0) and $AuCl_3$ when in contact with [NEt_3Me]Cl.

In addition, we recognized the formation of large, insoluble, golden particles in the bichloride (**Figure S4**). We separated these particles of the remaining residue and measured again a XRPD pattern (**Figure S9**). In this case, a clear assignment of the signals to Au(0) is possible and is in good agreement with the literature.[6-8]

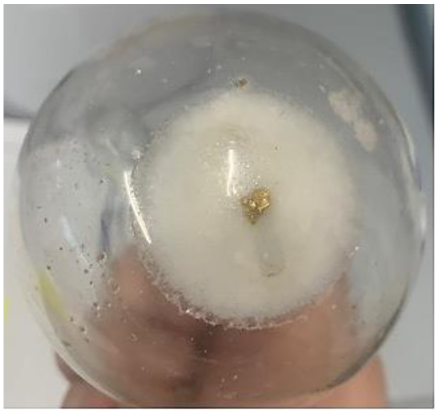

Figure S4 Formation of large, insoluble, golden particles in the bichloride during the hydrochlorination of acetylene using the bichloride-based Ionic Liquid $[NEt_3Me][Cl(HCl)_{2.5}]$ and AuCl.

Solubility of acetylene in $[NEt_3Me][Cl(HCl)_{2.5}]$

The solubility of acetylene in the bichloride-based Ionic Liquid $[NEt_3Me][Cl(HCl)_{2.5}]$ at room temperature was determined by putting a defined amount with a defined vapor pressure of acetylene above the bichloride. A decrease of the vapor pressure was observed, and the quantity of the dissolved acetylene was determined gravimetrically (3.9 g L^{-1}, 0.1507 mol L^{-1}).

Catalyst characterization

X-ray absorption spectroscopy (XAS) measurements were performed at the Swiss-Norwegian beamlines (SNBL, BM31) at the European Synchrotron Radiation Facility (ESRF). The X-ray beam was collimated using a double-crystal liquid nitrogen-cooled Si(111) monochromator and calibrated using Pd foil.[9] The gas filling inside the ionization chambers ensured optimal absorption at the Pd *K* absorption edge. All spectra were recorded in transmission mode at room temperature, using a one-element silicon drift detector with Peltier cooling. Continuous scanning was performed for the Pd *K* edge (between 24.15 and 25.32 keV), and the step size was set to 0.5 eV, with a scan duration of 150 s. The incident X-ray beam was focused on a 3 mm (horizontal) by 0.2 mm (vertical) area. The resulting spectra were energy calibrated, background corrected, normalized, and analyzed using the Demeter software package.[10] k^3-weighted extended X-ray absorption fine structure (EXAFS) spectra were fitted in the optimal k-space (3-11 Å^{-1}) and R-space (1-3 Å) windows. An amplitude reduction factor of 0.78 was determined by fitting the EXAFS spectrum of a Pd foil (**Table S1**).

Table S1 Fitting parameters derived from the Pd K edge EXAFS spectra of selected catalysts. [a]Coordination number; [b]Debye-Waller factor, [c]Coordination shell distance; [d]*R*-factor

**Catalyst**	**Coordination**	**CN[a] / -**	**$\sigma^{2[b]}$ / Å²**	***R*[c] / Å**	***Rf*[d] / -**
$PdCl_2$	Pd-Cl	4.6 ± 0.2	0.003 ± 0.002	2.31 ± 0.03	0.008
$PdCl_2$+[NEt_3Me][Cl(HCl)$_n$]	Pd-Cl	3.7 ± 0.2	0.003 ± 0.002	2.31 ± 0.03	0.009
$PdCl_2$+[NEt_3Me][Cl(HCl)$_n$]-1c	Pd-Cl	4.8 ± 0.2	0.003 ± 0.002	2.32 ± 0.03	0.005
$PdCl_2$+[NEt_3Me][Cl(HCl)$_n$]-10c	Pd-C	4.1 ± 0.2	0.003 ± 0.002	2.32 ± 0.03	0.013
	Pd-Cl	0.6 ± 0.2	0.003 ± 0.002	2.05 ± 0.03	
[NEt_3Me]$_2$[$PdCl_4$]	Pd-Cl	4.5 ± 0.2	0.003 ± 0.002	2.31 ± 0.03	0.006
[NEt_3Me]$_2$[$PdCl_4$]-1c	Pd-Cl	4.1 ± 0.2	0.003 ± 0.002	2.32 ± 0.03	0.025

IR Spectra

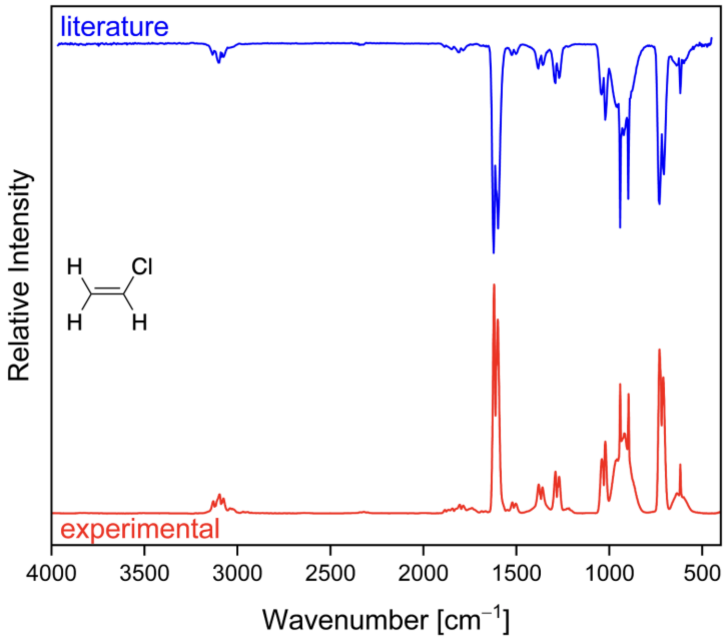

Figure S5 IR spectrum of the purified product (VCM) of the reaction of acetylene with [NEt_3Me][Cl(HCl)$_{2.5}$] (red) and the corresponding literature spectrum of VCM (blue).[5]

VCM: IR (FTIR), $\tilde{\nu}$ = 3129, 3095, 3075, 1884, 1844, 1804, 1786, 1622, 1598, 1521, 1504, 1381, 1358, 1289, 1269, 1041, 1022, 963, 942, 923, 898, 730, 715, 636, 618, 603 cm^{-1}.

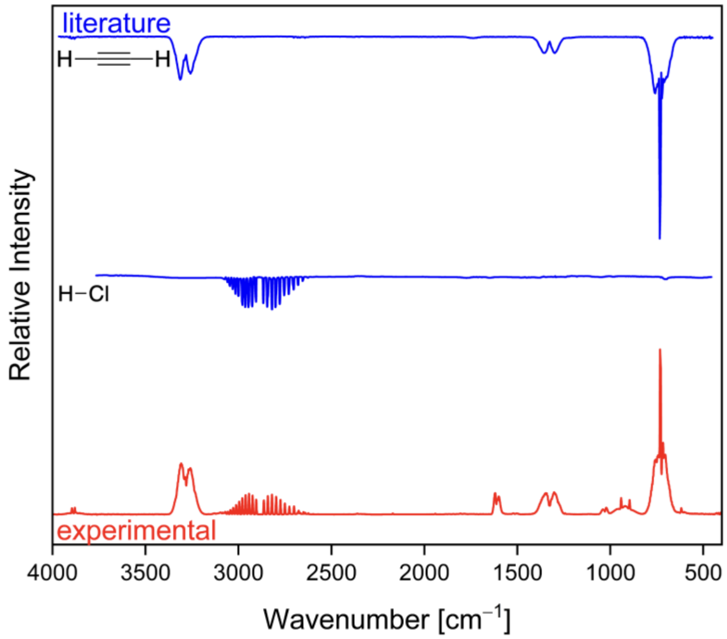

Figure S6 IR spectrum of the purified product mixture (HCl and acetylene) of the reaction of acetylene with [NEt_3Me][Cl(HCl)$_{2.5}$] (red) and the corresponding literature spectrum of HCl and acetylene (blue).[5]

HCl: IR (FTIR), $\tilde{\nu}$ = 3043, 3028, 3013, 2996, 2979, 2962, 2943,2924, 2905, 2863, 2842, 2820, 2797, 2774, 2750, 2726, 2701 cm^{-1}.

C_2H_2: IR (FTIR), $\tilde{\nu}$ = 3896, 3880, 3308, 3259, 1619, 1600, 1347, 1302, 1022, 942, 896, 730, 716, 618 cm^{-1}.

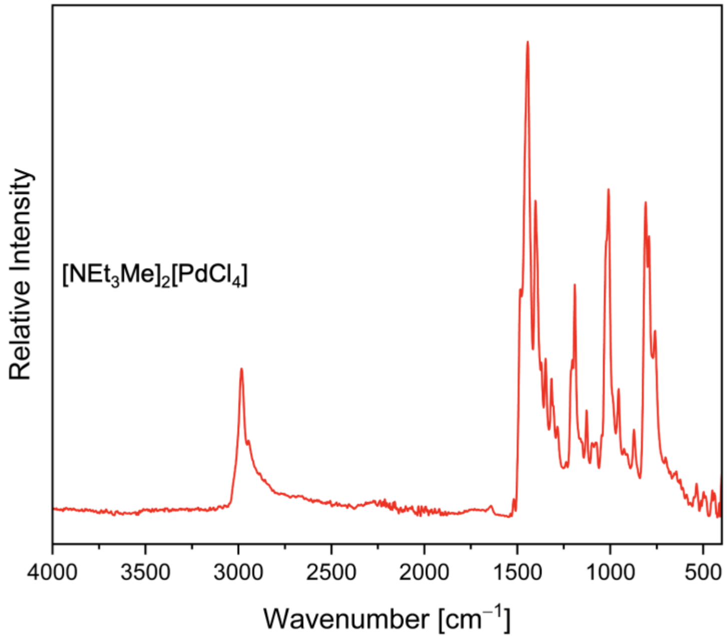

Figure S7 IR spectrum of [NEt_3Me]$_2$[$PdCl_4$].

Powder Diffractometry

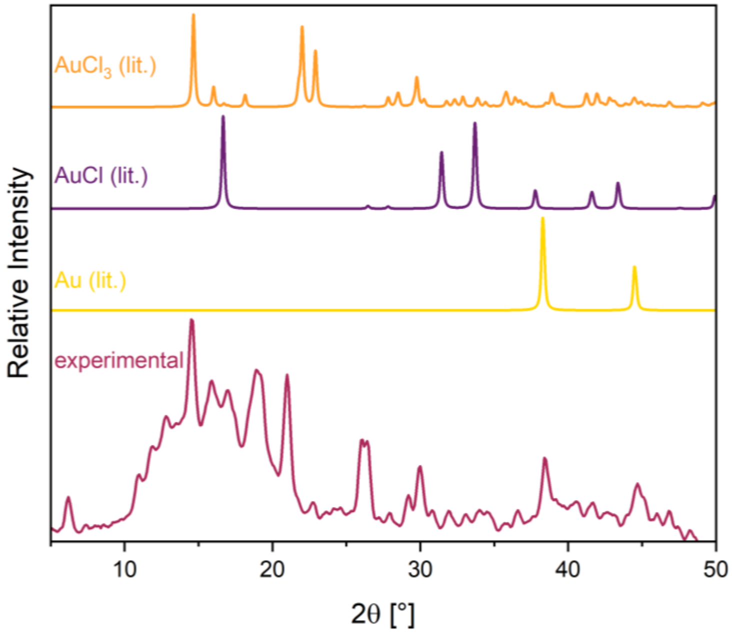

Figure S8 X-ray powder diffraction pattern of an experimental mixture of $[NEt_3Me]Cl$ and AuCl under inert conditions (pink) and the corresponding literature spectra of Au (yellow),[6] AuCl (purple),[7] and $AuCl_3$ (orange).[8]

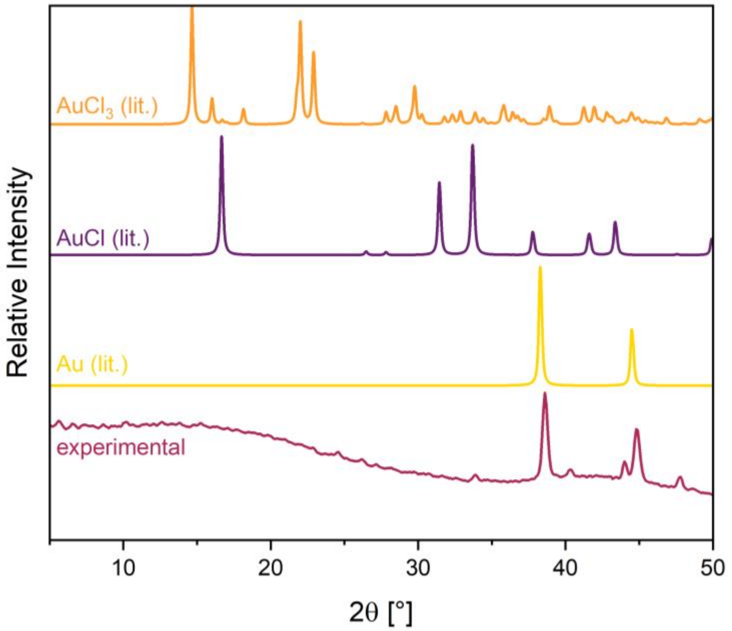

Figure S9 X-ray powder diffraction pattern of the large, insoluble, golden particles formed by the experimental hydrochlorination of acetylene using $[NEt_3Me][Cl(HCl)_{2.5}]$ and AuCl (pink) and the corresponding literature spectra of Au (yellow),[6] AuCl (purple),[7] and $AuCl_3$ (orange).[8]

Molecular structures in the solid state

$[NEt_3Me]_2[PdCl_4]$

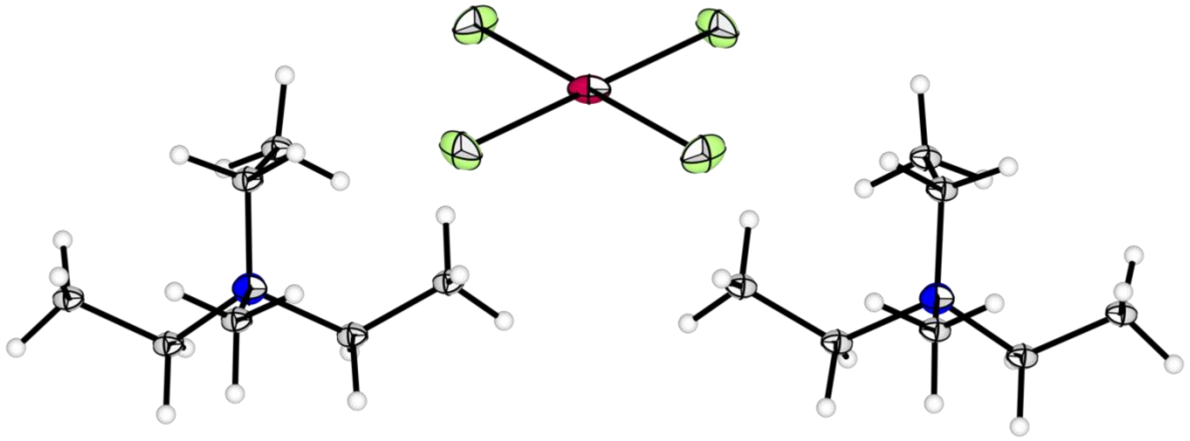

Figure S10 Molecular structure in the solid state of $[NEt_3Me]_2[PdCl_4]$. Displacement ellipsoids set at 50% probability.

Crystal data

Table S2 Crystal data and structure refinement for $[NEt_3Me]_2[PdCl_4]$

**Empirical formula**	$[NEt_3Me]_2[PdCl_4]$
**Formula weight**	480.65
T **[K]**	100.00
**Crystal system**	orthorhombic
**Space group**	*Immm*
a **[Å]**	9.1525(11)
b **[Å]**	9.8875(11)
c **[Å]**	11.7241(14)
α **[°]**	90
β **[°]**	90
γ **[°]**	90
V **[Å^3]**	1061.0(2)
**Z**	2
ρ_{calc} **[g cm^{-3}]**	1.505
μ **[mm^{-1}]**	1.375
**F(000)**	496.0
**Crystal size [mm^3]**	0.114 × 0.113 × 0.108
**Radiation**	MoK_α (λ = 0.71073)
**2Θ range for data collection [°]**	5.39 to 52.804
**Reflections collected**	642
**Independent reflections**	642 [R_{int} = 0.0669, R_{sigma} = 0.0184]
**Data/restraints/parameters**	642/72/45
**Goodness-of-fit on F^2**	1.187
**Final *R* indexes [I>=2σ (I)]**	R_1 = 0.0584, wR_2 = 0.1177
**Final *R* indexes [all data]**	R_1 = 0.0657, wR_2 = 0.1216
**Largest diff. peak/hole [e Å^{-3}]**	1.03/–1.43
**CCDC deposition number**	2410263

Computational details

All calculations were performed using the TURBOMOLE program code, Version 7.8.[11,12] Structures were optimized using the r^2SCAN-3c composite approach, the corresponding basis sets, and default numerical settings.[13] Solvation effects on the structures were included using the COSMO approach[14] with an infinite dielectric constant. All structures were identified as minima and first order transition states, respectively, by the appropriate number of vibrational modes with imaginary frequencies. Free energies for all optimized structures were calculated from gas-phase single point energies at the r^2SCAN-3c level, augumented by thermal contributions from the standard rigid-rotor-harmonic-oscillator treatment and free energies of solvation obtained at the COSMO-RS level[15-17] using $[NEt_3Me][Cl(HCl)_2]$ as a solvent. COSMO-RS calculations were performed with COSMOtherm program version C30_1201 and a BP-TZVPD-FINE level parameterization (BP_TZVPD_FINE_HB2012_C30_1201).

Cartesian coordinates

Given below are the optimized structures (at r^2SCAN-3c level) of the chemical species involved in the reaction (numbers refer to **Figure 3** of the main text).

Substrate 1

Pd	0.4714153	0.2502330	0.0000000
Cl	–0.9873145	1.0549984	1.6590392
Cl	–0.9873145	1.0549984	–1.6590392
Cl	1.9249312	–0.5483345	1.6631894
Cl	1.9249312	–0.5483345	–1.6631894
H	–2.2915208	–0.8336821	0.0000000
C	–2.9040469	–1.7122639	0.0000000
C	–3.5861758	–2.7047235	0.0000000
H	–4.1923100	–3.5833024	0.0000000

TS 1

Pd	–0.8618203	0.0796723	0.0588025
Cl	–0.6138468	1.8256018	1.6054438
Cl	–2.0064385	–1.2823407	1.8027357
Cl	–1.6523977	1.7317821	–1.6725668
Cl	–1.1533936	–1.6645802	–1.4820429

H	1.6249716	–1.2627243	1.1859945
C	1.5061765	–0.5625844	0.3882822
C	1.5086005	0.2293899	–0.5358885
H	1.6481473	0.9057814	–1.3507604

Substrate 2

Pd	–0.3980012	0.1126766	–1.0438725
C	0.5890217	–0.1974446	0.8663051
C	1.4034332	–0.5049234	–0.0068520
Cl	–1.1640733	–2.1011856	–1.0169060
Cl	–1.9845605	0.6392658	–2.6993685
Cl	0.3690102	2.3266358	–1.0755018
H	2.2644942	–0.8250671	–0.5597165
H	0.0422331	0.0207179	1.7796894
Cl	–1.1215574	0.5293247	3.7562228

TS 2

Pd	-1.2822101	-0.2646947	0.0272993
C	1.1727816	-0.1442693	–0.0320705
C	0.4493748	0.8709926	0.0071493
Cl	–1.3244237	–0.2025438	–2.3213160
Cl	–3.4633305	–1.2404428	0.0628640
Cl	–1.2151110	–0.2766154	2.3761749
H	0.4279388	1.9471769	0.0303984
H	1.5250837	–1.1515800	–0.0619659
Cl	3.7098964	0.4619765	–0.0885334

Product 2

C	–0.0203352	–0.9397717	1.3068659
Cl	0.8092433	–2.1444625	2.3774456
C	0.6006902	–0.3962658	0.2723758
Pd	0.2503535	0.8529137	–0.9996371
Cl	–1.2749475	2.3820091	–2.6738943
Cl	–1.3129402	2.0180086	0.7498332

Cl	0.8491753	–0.3397844	–2.7259197
H	1.6277172	–0.6812102	0.0383227
H	–1.0282497	–0.7514368	1.6546079

Substrate 3

C	0.4072851	–0.8196148	1.5044241
Cl	1.2550336	–1.9795847	2.5969461
C	1.0314286	–0.2509933	0.4880703
Pd	0.1630562	0.9880265	–0.7968467
Cl	–0.8224774	2.5363954	–2.4634029
Cl	–0.8395048	2.1843380	0.9581649
Cl	1.2340266	–0.2313346	–2.5129007
H	2.0759851	–0.4786622	0.2727113
H	–0.6210665	–0.6774178	1.8130316
H	–1.3880771	–0.2384851	–0.8808967
Cl	–2.4956892	–1.0326675	–0.9793014

TS 3

C	1.6233060	0.2541314	–0.3293654
Cl	3.3009847	–0.1347168	0.0643049
C	0.5935840	–0.2726190	0.3520474
Pd	–1.2571082	–0.4559306	–0.5342619
Cl	–3.3258262	–0.9096851	–1.6758216
Cl	–0.9983980	1.5302145	–1.7753883
Cl	–1.5259437	–2.4162426	0.7440097
H	0.8194231	–1.0069501	1.1267468
H	1.5518524	0.9821175	–1.1298701
H	–0.1456733	0.7069127	0.9991321
Cl	–0.6362019	1.7227690	2.1584663

Substrate 4

Pd	0.0687329	–0.1627863	–0.6615700
C	0.1010831	0.3919289	1.4617664
C	1.3877542	0.3999994	0.9484188

Cl	0.0629178	–2.3930183	0.0649350
Cl	–0.7930483	–0.8597794	–2.7525341
Cl	0.1998216	2.0293810	–1.4850971
H	1.8891735	1.3320647	0.7078700
H	–0.3344569	–0.4575266	1.9915047
Cl	–1.4204586	–1.5181652	4.0162456
H	2.0009332	–0.4865456	1.0908546
Cl	–0.7566786	1.8759300	1.8363607

TS 4

C	–0.1100859	1.1568909	0.8365645
Cl	–1.4275311	2.2569912	0.4610842
C	0.9333909	0.9675588	–0.0006661
Pd	–0.3137357	–1.0720034	–0.3886007
Cl	–2.5477105	–1.7299869	0.4714738
Cl	0.6943705	–2.0541180	1.4971424
Cl	–1.3215067	–0.1289350	–2.2784679
H	0.9955395	1.4689546	–0.9605628
H	–0.1617878	0.7703004	1.8482104
H	1.8077997	0.4392098	0.3663522
Cl	1.4512560	–2.0748615	–1.8525310

Product 4

C	1.5251187	2.5545989	0.5438789
Cl	0.3968006	3.7639696	–0.0669158
C	1.9135926	1.5223954	–0.1862206
Pd	–0.2447572	–0.7511869	–0.0073516
Cl	–1.8645732	0.4299395	1.2217823
Cl	0.6590743	–1.5994800	1.9937701
Cl	–1.1680897	0.0647439	–2.0128395
H	1.5693683	1.3572136	–1.2010115
H	1.8253644	2.7489381	1.5678238
H	2.5950750	0.7991908	0.2510080
Cl	1.3373450	–1.9879314	–1.2419098

Substrate 1 (Pt)

Pt	0.4721071	0.2853900	0.0002003
Cl	–1.0060987	1.0569855	1.6648091
Cl	–1.0052388	1.0568797	–1.6652782
Cl	1.9486773	–0.4822832	1.6641306
Cl	1.9503789	–0.4810300	–1.6627855
H	–2.1213222	–1.0141946	–0.0029184
C	–2.8444992	–1.8050414	–0.0015585
C	–3.6521074	–2.6984137	0.0007412
H	–4.3693017	–3.4887033	0.0026592

TS 1 (Pt)

Pt	–0.9022998	0.1039289	0.0371068
Cl	–0.6640369	1.8701201	1.5909193
Cl	–1.9450604	–1.2937644	1.8093529
Cl	–1.5359469	1.7394888	–1.7158073
Cl	–1.2035649	–1.6570977	–1.5114091
H	1.7553740	–1.2981390	1.1778623
C	1.5619130	–0.5904091	0.4029687
C	1.3679046	0.2244563	–0.4924052
H	1.5657163	0.9014141	–1.2985884

Substrate 2 (Pt)

Pt	–0.3971758	0.1148704	–1.0801552
C	0.5129866	–0.1570945	0.8399115
C	1.3566613	–0.4781255	–0.0166046
Cl	–1.1908035	–2.0998743	–1.0232083
Cl	–1.9448918	0.6165852	–2.7895306
Cl	0.3882926	2.3323073	–1.1506762
H	2.2712390	–0.8136427	–0.4671079
H	0.0170267	0.0407019	1.7852135
Cl	–1.0133351	0.4442722	3.9021577

Substrate 1 (Au)

Au	0.1111033	–0.0352346	0.0000000
Cl	–0.6007884	1.4529018	1.6487786
Cl	–0.6007884	1.4529018	–1.6487786
Cl	0.8556060	–1.5102472	1.6464195
Cl	0.8556060	–1.5102472	–1.6464195
H	–3.8965165	–0.5870735	0.0000000
C	–3.2048118	–1.3993022	0.0000000
C	–2.4226934	–2.3139895	0.0000000
H	–1.7241215	–3.1201204	0.0000000

TS 1 (Au)

Au	0.0945456	–0.0016504	0.0183449
Cl	0.1200927	2.3316015	0.0910969
Cl	–0.6310893	–0.0524808	2.3345900
Cl	–1.7090147	0.0603375	–1.8199878
Cl	0.0844444	–2.3356612	–0.0239016
H	3.1213719	–0.0232798	0.3085750
C	2.4860332	–0.0024420	–0.5529843
C	1.8544558	0.0232215	–1.5977643
H	1.4742713	0.0459535	–2.5986556

Substrate 2 (Au)

Au	–0.5037696	0.1625239	–0.5543610
C	1.0545156	–0.3520080	1.0016885
C	1.6010378	–0.5638859	–0.0774236
C	–1.2303336	–2.0643561	–0.5821776
Cl	–2.2374661	0.7359772	–2.0222134
Cl	0.3343283	2.3445658	–0.7094967
H	2.2160712	–0.7945356	–0.9257169
H	0.7311027	–0.2196429	2.0158619
Cl	–1.9654864	0.7513616	1.8538388

References and notes

[1] G. M. Sheldrick, *Acta Cryst. A* **2015**, *71*, 3–8.

[2] G. M. Sheldrick, *Acta Cryst. C* **2015**, *71*, 3–8.

[3] O. V. Dolomanov, L. J. Bourhis, R. J. Gildea, J. A. K. Howard, H. Puschmann, *J. Appl. Cryst.* **2009**, *42*, 339–341.

[4] G. H. Dreyhsig, P. Voßnacker, M. Kleoff, H. Baunis, N. Limberg, M. Lu, R. Schomäcker, S. Riedel, *Sci. Adv.* **2014**, *10*, eadn5353.

[5] NIST Chemistry Webbook, Sadtler Research Labs Under US-EPA Contract, 3rd December 2024; https://webbook.nist.gov.

[6] L. P. Salamakha, E. Bauer, S. I. Mudryi, A. P. Gonçalves, M. Almeida, H. Noël, *J. Alloys Compd.* **2009**, *479*, 184–188.

[7] E. M. W. Janssen, J. C: W. Folmer, G. A. Wiegers, *J. Less-common Met.* **1974**, *38*, 71–76.

[8] P. Schwerdtfeger, P. D. W. Boyd, S. Brienne, A. K. Burrell, *Inorg. Chem.* **1992**, *31*, 3411–3422.

[9] W. van Beek, O. V. Safonova, G. Wiker, H. Emerich, *Ph. Transit.* **2011**, *84*, 726–732.

[10] M. Newille, *J. Synchrotron Radiat.* **2001**, *8*, 322–324.

[11] TURBOMOLE V7.8 2023, a development of University of Karlsruhe and Forschungszentrum Karlsruhe GmbH, 1989-2007, TURBOMOLE GmbH, since 2007; available from *https://www.turbomole.org*. (last accessed Dec. 05, 2024)

[12] S. G. Balasubramani, G. P. Chen, S. Coriani, M. Diedenhofen, M. S. Frank, Y. J. Franzke, F. Furche, R. Grotjahn, M. E. Harding, C. Hättig, A. Hellweg, B. Helmich-Paris, C. Holzer, U. Huniar, M. Kaupp, A. Marefat Khah, S. Karbalaei Khani, T. Müller, F. Mack, B. D. Nguyen, S. M. Parker, E. Perlt, D. Rappoport, K. Reiter, S. Roy, M. Rückert, G. Schmitz, M. Sierka, E. Tapavicza, D. P. Tew, C. van Wüllen, V. K. Voora, F. Weigend, A. Wodyński, J. M. Yu, *J. Chem. Phys.* **2020**, *152*, 1841087.

[13] S. Grimme, A. Hansen, S. Ehlert, J.-M. Mewes, *J. Chem. Phys.* **2021**, *152*, 064103.

[14] A. Klamt, G. Schüürmann, *J. Chem. Soc., Perkin Trans. 2* **1993**, *2*, 799–805.

[15] F. Eckert, A. Klamt, *AIChE. J.* **2002**, *48*, 369–385.

[16] A. Klamt *Wiley Interdiscip. Rev. Comput. Mol. Sci.* **2011**, *1*, 699–709.

[17] A. Hellweg, F. Eckert, *AIChE. J.* **2017**, 63, 3944–3954.
